# Equine odontoclastic tooth resorption and hypercementosis (EOTRH): microspatial distribution of trace elements in hypercementosis-affected and unaffected hard dental tissues

**DOI:** 10.1038/s41598-023-32016-6

**Published:** 2023-03-28

**Authors:** Alexandra L. Wright, Edward T. Earley, Christine Austin, Manish Arora

**Affiliations:** 1 Department of Clinical Sciences, College of Veterinary Medicine, Cornell University, 930 Campus Rd, Ithaca, NY 14850 USA; 2 Environmental Medicine and Public Health, Icahn School of Medicine at Mount Sinai, 1 Gustave L Levy Place, New York, NY 10029 USA; 3 Institute for Exposomic Research, Icahn School of Medicine at Mount Sinai, New York, NY USA

**Keywords:** Pathogenesis, Dental diseases

## Abstract

Equine Odontoclastic Tooth Resorption and Hypercementosis (EOTRH) is a common, painful and poorly understood disease. Enamel, dentin and cementum accumulate both essential and toxic trace elements during mineralization. Characterization of the spatial accumulation pattern of trace elements may provide insight into the role that toxic elements play and inform biological processes affecting these hard dental tissues for future research. Laser ablation-inductively coupled plasma-mass spectrometry (LA-ICP-MS) was used to map the distribution of multiple trace elements and heavy metals across equine healthy and diseased (hypercementosis-affected) hard dental tissues among four teeth extracted from horses with EOTRH. Results showed banding patterns of some trace elements (lead, strontium, barium), reflecting the temporal component of accumulation of trace elements during dentin mineralization. Essential elements zinc and magnesium did not show banding patterns. Comparison to the unaffected cementum and dentin adjacent to the hypercementosis region showed that there is an underlying incremental pattern in the uptake of some metals with spatial irregularities. This supports a possible metabolic change involved in hypercementosis lesion development. This represents the first use of LA-ICP-MS to study the microspatial distribution of trace elements in equine teeth, establishing a baseline for elemental distribution in normal and EOTRH impacted dental hard tissue.

## Introduction

Disorders of tooth mineralization can cause chronic pain and deformities affecting appetite and general health. Teeth are composed of a number of essential minerals including calcium, manganese, zinc and magnesium^1,2^. These and other trace minerals are essential nutrients, and optimal maintenance of body stores is important for health and performance in horses, including oral health. During tooth development, ameloblasts facilitate ion transport that allows hydroxyapatite crystal formation^1,3^. The hydroxyapatite crystals in enamel contain many trace elements acquired through the process of mineralization and maturation. Dentin has a similar accumulation of trace elements over time, as secondary dentin is continually formed and mineralized post-eruption as long as the tooth remains vital^1,4^. Cementum, the final mineralized dental tissue that is the peripheral layer of the root, goes through a similar biomineralization process when forming and establishing its attachment to the periodontal ligament^5^. Thus, trace essential elements play an important role in tooth development in mammals, forming a solid mineral matrix as they are incorporated into dental hard tissues during mineralization^1,3,4,6^. Toxic elements, particularly heavy metals, have chemical properties similar to some trace elements, so they can also be incorporated into the tooth matrix, potentially disrupting optimal mineralization.

In humans, metabolic disorders that alter the natural body balance of essential elements can cause dental pathologies^1,2,7^. Calcium, phosphate, and magnesium^2^ play an important role in the remineralization and demineralization of enamel. Beyond accumulation in dental tissues during tooth formation, elements are also involved in interactions involving shared transport pathways. For example, lead and zinc ions are known to compete with calcium and phosphate ions, thus creating an offset in normal concentrations of these essential elements in teeth and disrupting the architecture^1,7,8^. When incorporation of these essential elements is disrupted, by changes in zinc in saliva for example, dental pathology such as carious lesions can result^7^. Toxic metals have also been associated with dental caries and periodontal disease^2,7,9–13^. Studies link exposure to lead and cadmium with increased prevalence of pediatric dental caries^9,11^. Notably, recent studies have demonstrated significant differences in concentrations of essential elements in oral fluid when comparing subjects with periodontitis groups to healthy groups in humans, highlighting the need for further research focused on the influence of trace elements on oral health^12,13^.

Research related to equine oral health has received increasing attention in veterinary medicine over the last two decades. In particular, Equine Odontoclastic Tooth Resorption and Hypercementosis (EOTRH) has been a growing topic of interest, since its first description in 2004^14–18^. This progressive and painful disease is common among aged horses and can cause oral pain, tooth fractures and tooth loss, periodic inappetence, and weight loss in severe cases^19^. Histopathologic reports describe early signs involving widening of the periodontal ligament and osteolysis. This is followed by odontoclastic activity (tooth resorption) of the cementum, enamel and dentin, and in some cases leading to hypercementosis at the roots of the incisors^14,16,17^. The high incidence and associated morbidity of the disease among aging horses makes further understanding of this disease crucial for veterinary practitioners, in particular because the factors contributing to the etiology of EOTRH remain poorly understood.

Pasture grazing time and certain dietary factors that may contribute to EOTRH have been proposed as risk factors, given differences in calcium and phosphorous content that occur in grazing (e.g., alfalfa and other legumes higher in calcium compared with grasses lower in calcium content)^20^. Furthermore, horses can be exposed to toxic metals through environmental contamination of soil or water (e.g., via grazing or water sources) as well as metal-containing compounds in therapeutics or dietary products. These issues have led to a growing interest in development methodologies to study exposure to trace elements and their health impacts^21,22^. The role of toxic elements in equine dental health remains largely unknown. Characterizing the distribution of these elements within dental hard tissues, as well as the disruption of the microspatial patterning of essential and toxic trace elements in diseased compared to normal hard dental tissues, may provide insights into the pathophysiological mechanisms related to disease processes. As dental hard tissues are relatively stable, the metals deposited in teeth during mineralization are to a large extent retained. Thus, teeth can provide a permanent, cumulative, and quantitative record of insults related to environmental pollutants including heavy metals as well as deposition of other trace elements. While novel methods to examine the spatial distribution of trace elements in teeth has been applied in rodent, human and primate teeth^8,23–26^. no prior study has examined uptake and spatial distribution of trace elements in equine dental tissues. This study is the first to report of the use of laser ablation-inductively coupled plasma-mass spectrometry (LA-ICP-MS) to map the distribution of multiple trace elements and heavy metals across equine enamel, dentin, and cementum in healthy and diseased (hypercementosis-affected) hard dental tissues in teeth extracted from horses with EOTRH.

## Methods

### Ethics and inclusion statement

Ethical review and approval was not required for the animal study because the study was not experimental and only used discarded tissues obtained from routine veterinary care. Assays were conducted in discarded teeth that had been extracted based on standard medical indications from horses presenting for health care to an authorized, licensed veterinarian. This situation is not regarded as animal experimentation according to the Animal Experimentation Act. The study and its design was also discussed with the Cornell University Veterinary Clinical Studies Committee (CUVCSC) and an IACUC exemption was obtained (Protocol ID#: 030221-07).

### Study population and tooth extraction

Equine incisor teeth were included in this study from patients that had undergone incisor and canine tooth extraction for treatment of EOTRH. Teeth included in this study were extracted between May 2020 to May 2021 by a board certified equine veterinary dentist in standard technique^27^. All cases were diagnosed pre-operatively with intraoral radiography, as is standard of care. It was required that the teeth used for analysis were intact from crown to apex. Teeth were packaged individually, labeled by modified Triadan number, and assigned a study enrollment number to keep patient signalment and medical history blind to the individuals processing and analyzing all teeth.

### Tooth preparation and laboratory analysis for trace elements

**Figure 1:**
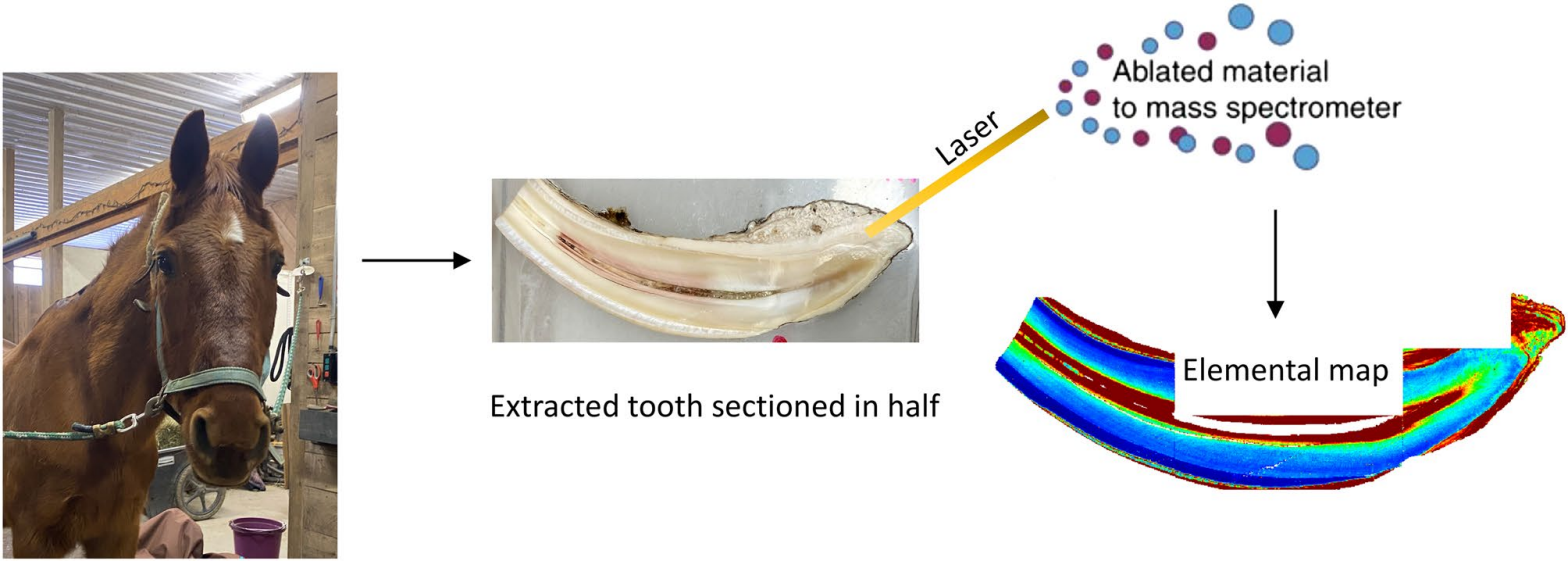
Overview of methods. Teeth were extracted from animals diagnosed with EOTRH. Teeth were sectioned, embedded in epoxy resin and polished to a flat surface. LA-ICP-MS was used to generate microspatial elemental maps to determine the distribution of trace elements across healthy unaffected tissue and areas affected by EOTRH.

Trace metal-free water produced through reverse osmosis (MilliQ water) was used to avoid external contamination during laboratory procedures and was used to clean the teeth. The teeth were then sectioned along the labiolingual plane using a slow speed rotary saw (IsoMet, Buehler) with a diamond tipped blade. One half of the tooth was embedded in epoxy resin and the cut surface polished down to 1 µm roughness with diamond paste (Fig. 1). An ESL NWR193 laser ablation system equipped with a Coherent ExciStar argon fluoride excimer laser was used. The laser ablation unit was connected to an Agilent Technologies 8800 triple-quadrupole ICP-MS by Tygon tubing. Details of this analytical methods have been published previously^8,28^.

**Figure 2:**
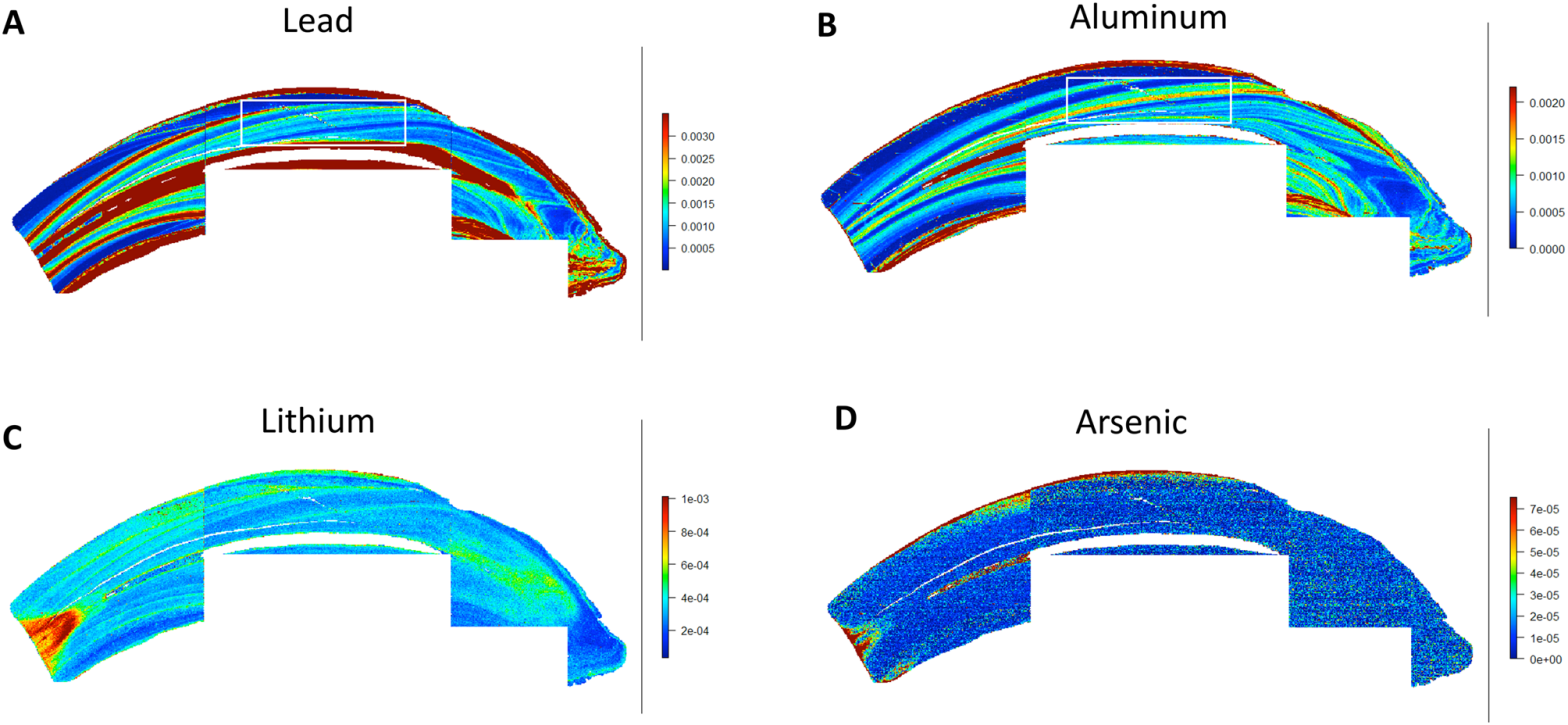
Toxic elements in equine teeth. Maps for lead (**A**), aluminum (**B**), lithium (**C**) and arsenic (**D**) are shown. Banding patterns are evident for lead and aluminum maps (i.e. alternating interspersed zones of high/low element concentration histologically – see white box). Orientation of the bands follows the developmental mineralization of enamel and dentin representing tooth maturation rings.

**Figure 3:**
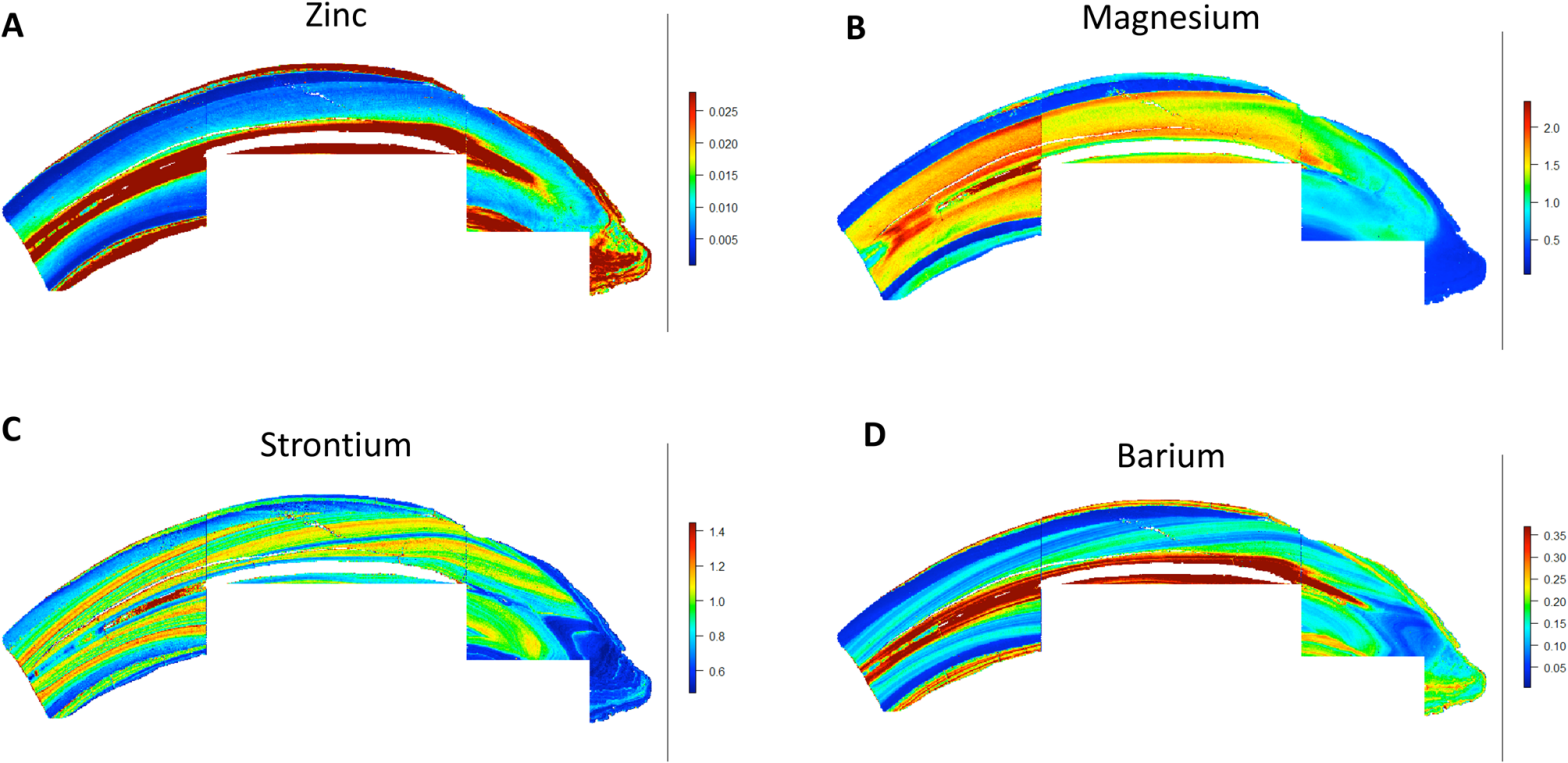
Essential and non-essential elements in equine teeth. Maps for zinc (**A**), magnesium (**B**), strontium (**C**), and barium (**D**) are shown. The essential elements zinc (**A**) and magnesium (**B**) did not show a banding pattern while non-essential elements, strontium (**C**) and barium (**D**) showed a banding pattern similar to that observed with lead.

**Figure 4:**
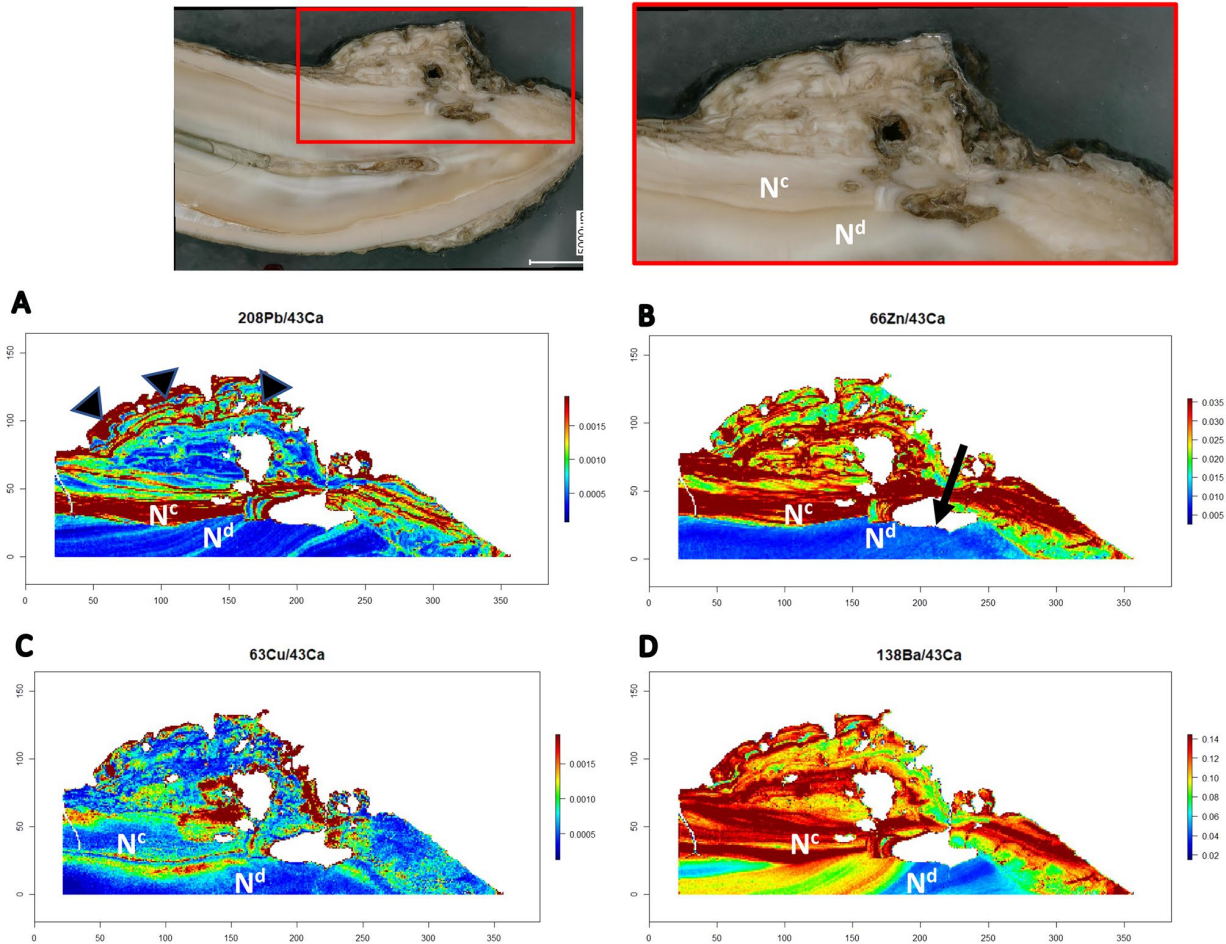
Elemental dysregulation in EOTRH. Maps of elemental distribution for hypercementosis lesions: lead (**A**), zinc (**B**), copper (**C**) and barium (**D**). Subsequent to establishing the distribution of trace elements in healthy unaffected tissue (Figs. 2 and 3), a region of hypercementosis (region outlined in red in top panels) was analyzed in the tooth shown in Figs. 1 and 2. Normal dentin marked with N^d^ and regular cementum marked with N^c^. Incremental markings of irregular cementum shown with black arrowheads on lead map. Tooth resorption lesions noted with black arrow on zinc map.

**Figure 5:**
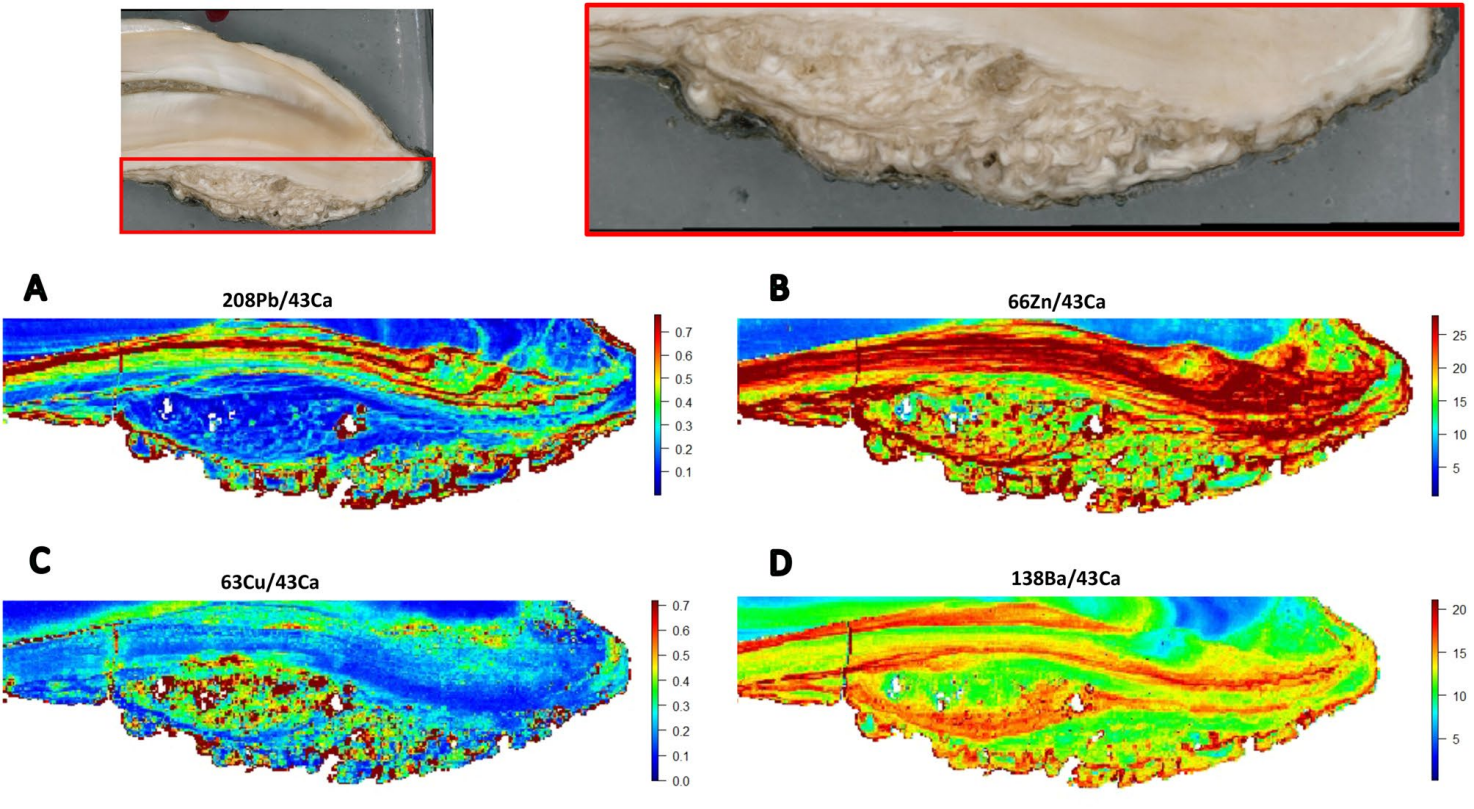
Second example of elemental dysregulation in EOTRH. Elemental maps of analyzed regions of a second tooth with hypercementosis (region outlined in red in top panels) is shown. Maps for lead (**A**), zinc (**B**), copper (**C**) and barium (**D**). Similar to the tooth shown in Fig. 4, there was hyperaccumulation of trace elements in the irregular hypercementosis-affected region and disrupted incremental growth patterns.

The LA-ICP-MS analysis was performed in two modes. First, for two teeth the entire cut surface was rastered, taking thousands of sampling points of linear scans adjacent to each another. Once these linear scans were combined, two-dimensional elemental maps were generated. The LA-ICP-MS analysis generates metal intensity data in .csv format which was converted into images by assigning a color relative to the metal ion intensity at each sampling point. The details of this method and the custom R code that was used to achieve this have been detailed previously^29^ The maps are shown in Figs. 2, 3, 4 and 5. A laser spot size of 80 µm, scan speed of 160 µm s^-1^, repetition rate of 60 Hz, laser power of 2.0 J cm^-1^ and ICP-MS total integration time of 0.5 s was used for imaging. Due to the large size of the teeth, smaller maps were run over multiple days. Subsequently, using this information as a guide single linear traces were taken from the coronal aspect of the tooth crown towards the apex. The results of these linear traces are shown in Supplemental Figures S1 and S2. A laser spot size of 35 µm, scan speed of 35 µm s^-1^, repetition rate of 40 Hz, laser power of 2.4 J cm^-1^ and ICP-MS total integration time of 1 s was used for the linear traces. In both modes, pre-ablation of the surface at a lower laser power (0.8 J cm^-1^) was used to minimize surface contamination. Metals were normalized to calcium to account for individual mineral density variation within and between samples and metal:Calcium ratios were corrected to NIST 610 to minimize sensitivity differences between days.

## Results

The LA-ICP-MS analysis scans teeth in the X–Y dimension to create two dimensional maps of multiple trace elements across the sectioned surface of the tooth covering the non-diseased (healthy) and diseased (hypercementosis-affected) regions in the same analytical scan. Overall, equine teeth showed clear and distinct variations in the distribution of different elements. Examples of eight elements that are known to either play key physiologic roles in dental histology or are known toxicants are provided. Mapping of both tooth-seeking elements (i.e. elements known to concentrate in teeth) such as lead, strontium, barium, magnesium, zinc and aluminum, and elements that do not bioaccumulate in teeth (arsenic and lithium) was performed. The concentration of these elements varied markedly as is evident from the intensity indices provided with each image panel in Figs. 2 and 3. Additional data are shown in Supplemental Information.

A clear banding pattern for lead, a known toxicant that is predominantly stored in teeth and bones following exposure, was noted in the dentin of the teeth (Fig. 2A). The banding pattern is formed due to higher levels of lead in dentin layers alternating with lower lead levels. Of importance is that these interspersed bands followed the developmental microanatomy of tooth mineralization, which these methods illustrate visually as incremental zones or maturation rings (additional details in Discussion). Lead also accumulated in the dentin immediately adjacent to the pulp. This pattern was also observed in two other toxic elements that were measured, namely lithium and aluminum. However, levels of arsenic were low and consistently close to the detection limit of the instrument (0.25 ug/g). No clear banding patterns were observed for arsenic.

In contrast to toxic metals, essential elements (zinc and magnesium) did not show a banding pattern (Fig. 3). Zinc levels were highest in the dentin immediately adjacent to the pulp. Levels were also high within the enamel and the peripheral cementum. Similarly, magnesium distribution did not vary markedly within the different dental tissues with slightly higher levels seen on the surface of enamel and very low levels in cementum compared to dentin. Overall, the data supports that magnesium and zinc distribution do not vary markedly over the course of enamel and dentin mineralization.

The non-essential elements strontium and barium showed a distribution similar to lead. Higher strontium and barium levels were distributed between areas of lower concentrations creating a banding pattern that was aligned with the incremental mineralization of teeth. Barium also showed higher accumulation adjacent to the pulp similar to what was seen for lead. These results were further confirmed by taking linear traces in two additional teeth from two different animals. The linear traces showed alternating high and low peaks in ion intensity which are shown in Supplemental Figures S1 and S2.

Hypercementosis affected tissue (irregular cementum) showed two major patterns that differed histologically from regular cementum and dentin (Figs. 4 and 5). First, hypercementosis-affected tissue had higher levels of some trace elements than dentin including lead, zinc, copper and barium. Other elements such as strontium and nickel were not different from dentin tissue. Second, the hypercementosis regions, while irregular in both gross appearance and the elemental maps, continued to show incremental markings (arrowheads in lead map of Fig. 4) with alternating levels of trace elements (lead and zinc), unlike the regular cementum that was more uniform. A clear boundary between healthy and hypercementosis regions could be seen in some elemental maps (see lead and zinc map in Fig. 4). In other cases, the transition between healthy and hypercementosis tissue was diffuse (see copper map in Fig. 4).

## Discussion

To date, little is known about the uptake and distribution of trace elements in equine teeth as research in this area has been sparse. One reason has been the lack of technologies that can quantify the histologic distribution of trace elements in dental tissues at micrometer resolution, giving a clearer picture of the developmental history of tooth formation, modeling and mineralization. This is first use of LA-ICP-MS to study the microspatial distribution of multiple essential and nonessential trace elements in equine teeth to establish a baseline for elemental distribution in dental hard tissue and to apply this approach to histologically study elemental distributions in EOTRH-affected dental tissue. This proof of concept study provides valuable insight on the spatial distribution of both essential and nonessential trace elements in equine dental tissue from EOTRH afflicted horses, comparing diseased areas and healthy, non-affected tissue in the same tooth as a control. Because teeth mineral rings are formed throughout tooth maturation, the approach illustrates the differences in mineralization over time in the same animal and illustrates the transition from normal mineralization to diseased mineralization. The results show clear differences in mineralization that vary based on the trace elements involved and may provide novel insight into the etiology of this chronic disorder. Trace element specific patterns were present in the healthy tissue with some elements showing periodic alterations between high and low levels as illustrated by the banding patterns discussed in the results. When measuring essential trace elements, the levels did not show such major fluctuations (e.g. zinc and magnesium), as expected in mineralization of normal tissue. The pattern of seeing banding for only non-essential trace elements is intriguing and deserves further study.

This is also the first study to use LA-ICP-MS to map a hypercementosis-affected region of a tooth in comparison to the healthy dental tissue regions of the same tooth, laying the ground work for future studies to compare disease-free and disease-affected animals. Hypercementosis is a common pathology observed in EOTRH, although it does not occur in every case^19^. Notable differences between hypercementosis-affected tissue compared with unaffected tissue were observed, including increased uptake of lead, zinc, copper and barium in the former. The hyperaccumulation of trace elements in the diseased tissue supports higher metabolism during cellular deposition of the protein matrix and then subsequent mineralization. Furthermore, the presence of incremental markings, albeit distorted, supports that this is a cellular process similar to the deposition of normal cementum but cellular control has been lost to some degree. This finding argues against a passive accumulation of mineral on the equine roots affected by EOTRH, as the areas of normal dental tissue did not show the same accumulation pattern. Equine cementum is different when compared to its brachydont counterpart. First, it covers both the periphery of the root as well as the clinical crown, and is also found within infundibula of certain teeth^30–32^. Additionally, cementum is highly cellular and more vascularized coronally along the root, as compared with the apex^32^. The hypercementosis sites in the teeth of this study were located in the apical third of the root, as is typical for this presentation of the disease. Since apical cementum is less vascularized than coronal cementum, blood level exposures cannot explain the accumulation of elements. Finally, hypercementosis is theorized to be a reparative mechanism in response to tooth resorption^15,18^, suggesting a cellular signaling pathway for disorganized production. The microspatial elemental distribution findings suggest that hypercementosis in EOTRH may have an altered growth rate component, in that the cells exhibit a higher metabolic rate than is normal. It is known that in benign tumors with altered growth rates, cells exhibit high metabolic rates that contribute to their local invasiveness and tissue proliferation^33,34^. Bioimaging of tissues with altered growth rate has shown that toxic elements accumulate at higher levels when compared to normal tissue^35–37^, as seen in the dental hard tissues of this study. The ‘metabolic rate hypothesis’ of hypercementosis proposed here deserves future research that may ultimately explore therapies that target the high metabolic rate of cells involved in hypercementosis in a manner similar to therapeutics targeting cancer cell metabolism^38^. Alternatively, the trace element accumulation observed may be due to other mechanisms such as higher metabolism from increased focal stress and biomechanical forces found in areas of initial resorptive lesions and hypercementosis^39–41^. Future investigation of the cellular metabolic rate may elucidate a better understanding of the biomechanical stress aged incisors undergo and how this may contribute to the etiology of the disease.

As teeth mature, many chemicals circulating in the blood are deposited in the mineralizing matrix, particularly trace elements, which are incorporated into the crystalline structure that preserves the timing and intensity of the trace element exposure^4,25^. The LA-ICP MS methods employed here have to date primarily been used to measure past chemical exposure in teeth in order to reconstruct associations between early life exposure and non-dental diseases^28,42–44^. Little research has considered the role of trace element deposition in dental diseases, with the exception of carious lesions in humans^45,46^. Therefore, this research methodology is an underutilized tool in the study of dental disease in human and veterinary medicine alike. Importantly, these methods can provide insight into the uptake of trace elements during mineralization. While this study focused on diseased teeth, the insights gleaned by establishing this analysis technique may also apply to healthy equine tooth development and other dental disease, both acquired and genetic. Furthermore, these results provide a novel hypothesis to study the underlying disease mechanisms in EOTRH-related hypercementosis. While the analysis allowed visualization of the diseased and normal portions of the teeth included, these results may not relate trace element accumulation patterns to the development of EOTRH in all cases, as the sample size is small. Different patterns might exist if more animals with EOTRH were studied. Additionally, this technology cannot measure trace elements in resorbed dental tissues since that tissue is no longer present. In vitro studies have been done to study cellular mechanisms involved in root resorption related to orthodontic tooth movement^47,48^, suggesting that future work could be done to establish an in vitro model to facilitate the study of cellular mechanisms involved in resorption from EOTRH. However, this work is a ground breaking introduction to the potential of LA-ICP-MS in equine health research, as it shows that patterns of deposition vary in EOTRH teeth and may be part of the underlying pathophysiology of the disease. By establishing this analytic method in equine teeth, this work will serve as the basis of multiple future studies on the role of trace elements in dental health and disease. Additional work is needed to uncover the cellular mechanisms underlying the hyperaccumulation of elements and dysregulated patterns of mineralization observed. Future studies including larger sample size as well as examining EOTRH diseased teeth of the different presentation types (tooth resorption predominant, hypercementosis predominant, or tooth resorption and hypercementosis combination) are needed to further characterize the disease and its link to toxic elements.

## Supplementary Information

Supplementary Information.

## Data Availability

Data are available on reasonable request to the corresponding author and pending clearances.
