# Supplementary material for: Equine odontoclastic tooth resorption and hypercementosis (EOTRH): microspatial distribution of trace elements in hypercementosis-affected and unaffected hard dental tissues
Source: Sci Rep. 2023 Mar 28;13:5048. doi: 10.1038/s41598-023-32016-6 (PMC10050172; doi:10.1038/s41598-023-32016-6)

Supplemental Material

Equine odontoclastic tooth resorption and hypercementosis (EOTRH): Microspatial distribution of elements in hypercementosis-affected and unaffected hard dental tissues

Alexandra L. Wright[a], Edward T. Earley[a], Christine Austin[b,c], Manish Arora[b,c]

[a]Department of Clinical Sciences, Cornell University, College of Veterinary Medicine, Ithaca, NY 14850

[b]Environmental Medicine and Public Health, Icahn School of Medicine at Mount Sinai, New York, NY, 10029

[c]Institute for Exposomic Research, Icahn School of Medicine at Mount Sinai, New York, NY

Corresponding Author

Alexandra Wright
930 Campus Rd
Ithaca, NY 14850
Email: aw656@cornell.edu
Tel: 607-253-3060

Figure S1. Aluminum (Al), lead (Pb), strontium (Sr) and barium (Ba) graphs are shown. Linear distribution of trace elements in healthy dental tissue in Horse ID: 132-102. Alternating periods of high (indicated as red arrow) and low intensity would appear as bands in elemental maps (as shown in Figures 2 and 3 in the main manuscript).

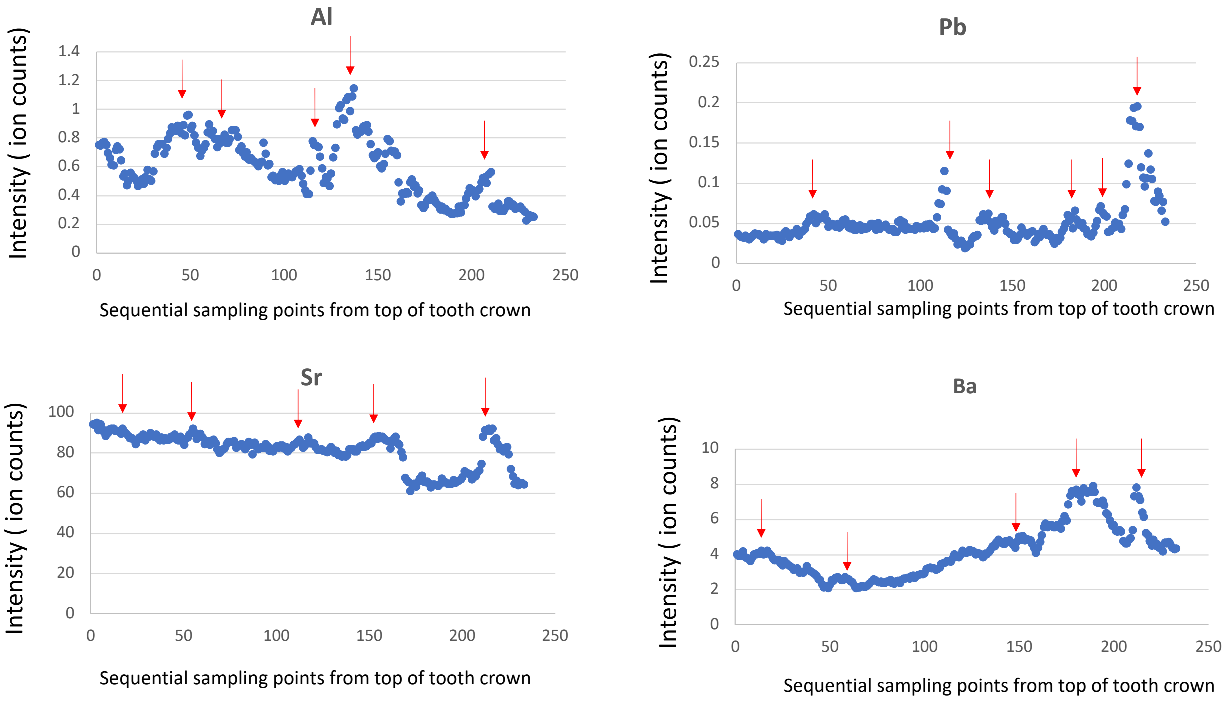

Figure S2. Aluminum (Al), lead (Pb), strontium (Sr) and barium (Ba) graphs are shown. Linear distribution of trace elements in healthy dental tissue in Horse ID: 108-302. Alternating periods of high (indicated as red arrow) and low intensity would appear as bands in elemental maps (as shown in Figures 2 and 3 in the main manuscript).

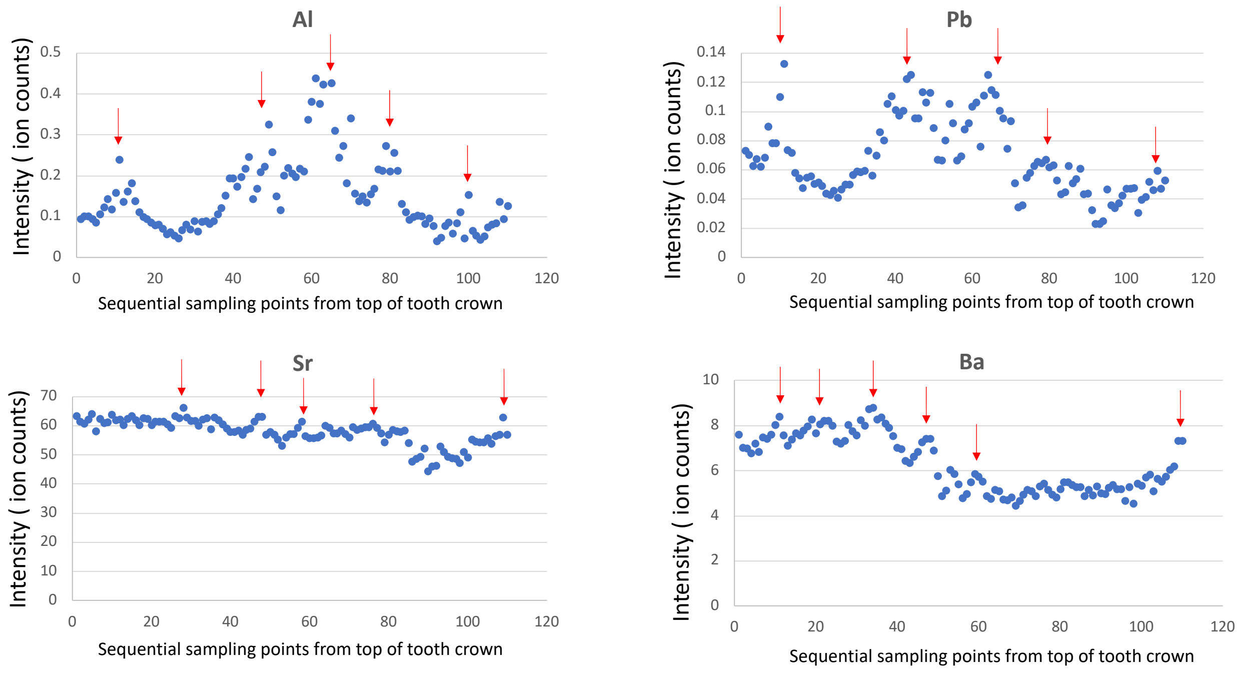

Supplement: Supplementary file 1 — Supplementary Information. [file 41598_2023_32016_MOESM1_ESM.pdf]
